# Long-term Health Outcomes of New Persistent Opioid Use After Gastrointestinal Cancer Surgery

**DOI:** 10.1245/s10434-024-15435-1

**Published:** 2024-05-18

**Authors:** Mujtaba Khalil, Selamawit Woldesenbet, Muhammad Musaab Munir, Muhammad Muntazir Mehdi Khan, Zayed Rashid, Abdullah Altaf, Erryk Katayama, Yutaka Endo, Mary Dillhoff, Susan Tsai, Timothy M. Pawlik

**Affiliations:** Department of Surgery, The Ohio State University Wexner Medical Center and James Comprehensive Cancer Center, Columbus, OH USA

**Keywords:** Opioids, Postoperative outcomes, Mortality, Healthcare utilization

## Abstract

**Background:**

New persistent opioid use (NPOU) after surgery has been identified as a common complication. This study sought to assess the long-term health outcomes among patients who experienced NPOU after gastrointestinal (GI) cancer surgery.

**Methods:**

Patients who underwent surgery for hepato-pancreato-biliary and colorectal cancer between 2007 and 2019 were identified using the Surveillance, Epidemiology, and End Results (SEER)-Medicare-linked database. Mixed-effect multivariable logistic regression and Cox proportional hazard models were used to estimate the risk of mortality and hospital visits related to falls, respiratory events, or pain symptoms.

**Results:**

Among 15,456 patients who underwent GI cancer surgery, 967(6.6%) experienced NPOU. Notably, the patients at risk for the development of NPOU were those with a history of substance abuse (odds ratio [OR], 1.45; 95% confidence interval [CI], 1.14–1.84), moderate social vulnerability (OR, 1.26; 95% CI, 1.06–1.50), an advanced disease stage (OR, 4.42; 95% CI, 3.51–5.82), or perioperative opioid use (OR, 3.07; 95% CI, 2.59–3.63. After control for competing risk factors, patients who experienced NPOU were more likely to visit a hospital for falls, respiratory events, or pain symptoms (OR, 1.45, 95% CI 1.18–1.78). Moreover, patients who experienced NPOU had a greater risk of death at 1 year (hazard ratio [HR], 2.15; 95% CI, 1.74–2.66).

**Conclusion:**

Approximately 1 in 15 patients experienced NPOU after GI cancer surgery. NPOU was associated with an increased risk of subsequent hospital visits and higher mortality. Targeted interventions for individuals at higher risk for NPOU after surgery should be used to help mitigate the harmful effects of NPOU.

**Supplementary Information:**

The online version contains supplementary material available at 10.1245/s10434-024-15435-1.

Opioid use in the United States (U.S.) has been declared a public health emergency amid a continuing rise in misuse, abuse, and fatal overdoses.^1,2^ In 2016 alone, more than 11.5 million people reported misuse of prescription opioids, and 1.9 million met the diagnostic criteria for prescription opioid abuse.^3^ Although the factors contributing to this public health issue are multifactorial, surgery plays a crucial role because a significant number of individuals receive their first opioid prescription during the perioperative period.^4,5^ Moreover, despite recent efforts aimed at mitigating over-prescription, the issue of excessive opioid prescription persists.^6^ Additionally, unused opioids are rarely discarded after surgery, creating a reservoir of pills for unintended use.^7^

Patients undergoing cancer surgery are more likely to be prescribed opioids due to the debilitating postoperative pain resulting from the underlying disease and surgical procedures.^8^ Moreover, individuals with cancer are distressed, undergo multiple invasive procedures, and may encounter uncoordinated prescriptions from multiple providers.^8^ In turn, patients with cancer are at a higher risk for the development of new persistent opioid use (NPOU) or opioid use disorder.^7^

NPOU occurs when a patient, initially opioid-naïve, is prescribed opioids for short-term postoperative pain relief.^7,9^ Subsequently, the patient continues to refill opioids beyond the expected period for the resolution of pain.^7,9^ Notably, NPOU is increasingly recognized as a serious and common surgical complication that can lead to chronic opioid abuse and fatal overdoses in the long term.^10,11^ For example, Santosa et al.^12^ reported that NPOU was associated with frequent emergency department visits, chronic opioid use, and a higher risk of mortality among patients undergoing common general surgical procedures.

Despite recent guidelines aimed at curbing opioid misuse, NPOU develops in up to 6% of patients undergoing common surgical procedures.^13^ Notably, patients undergoing cancer surgery often receive higher doses of opioids postoperatively and may face an increased risk for the development of NPOU.^8^ Nevertheless, the incidence, risk factors, and long-term health care outcomes of NPOU among patients undergoing gastrointestinal cancer surgery remain poorly defined. Therefore, the current study sought to evaluate the long-term health outcomes of NPOU among patients undergoing gastrointestinal cancer surgery using nationally representative data. In particular, we sought to characterize hospital visits due to falls, respiratory symptoms, and pain events, as well as 1 year mortality among individuals who experienced NPOU after gastrointestinal cancer surgery.

## Methods

### Data Source, Study Population, and Cohort Selection

The Surveillance, Epidemiology, and End Results (SEER)-Medicare-linked database was queried to identify patients who underwent surgery for gastrointestinal cancer. Data on cancer incidence is collected by SEER from 18 registries across 15 states, representing approximately 30% of the U.S. population.^14^ By matching 93% of Medicare beneficiaries’ medical claim files to individuals in SEER registries, the national Medicare health insurance program covers 97% of people age 65 years or older with cancer.^14^ The codes of the International Classification of Diseases, ninth and tenth editions (ICD-9/10), were used to identify patients ages 65 or older who underwent surgery for hepatopancreatobiliary (HPB) or colorectal cancer (CRC) between 2007 and 2019. If a patient underwent multiple eligible procedures, only the first surgical operation and postoperative period were included. Patients were excluded if they were not opioid-naive at the time of surgery or if the hospital stay for the index surgery exceeded 30 days. Additionally, patients were excluded if they were not discharged home or if they underwent a subsequent surgery requiring anesthesia within 12 months after the index surgery.

Opioid-naive was defined as no pharmacy-dispensing claim for any opioid during the 365 to 31 days before surgery.^12,15,16^ Any opioid prescription in the month before surgery was considered attributable to the procedure, accounting for preoperative prescriptions intended for postoperative use.^12,15,16^

**Fig. 1:**
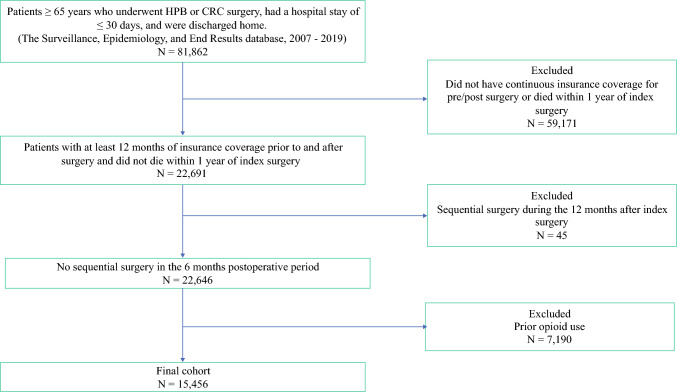
Flowchart to identify patients with new persistent opioid use (NPOU)

For the primary outcome, patients were required to maintain continuous enrollment in Medicare parts A, B, and D for 12 months before their surgical date and for 6 months afterward. For the secondary outcomes, patients were required to maintain continuous enrollment for 12 months both before and after their surgical date (Fig. 1). The Institutional Review Board at Ohio State University approved this study and waived the requirement for informed consent because the data were limited.

### Exposure

The exposure of interest was NPOU among opioid-naive patients. National Drug Codes were used to identify all Medicare part D claims for opioid prescriptions during the pre- and postoperative periods. In line with previous studies, NPOU was defined as an opioid prescription filled during both the first 90 day period (excluding the perioperative period of 30 days before surgery to 3 days afterward) and the subsequent 91 to 180 day period after surgery.^12,15,16^ Notably, normal surgical recovery is expected to occur within 90 days.^12,15^ This definition is more conservative than the 60 day threshold used by the International Association for the Study of Pain.^17^

### Covariates and Outcomes of Interest

Baseline covariates included patient age, sex, Charlson Comorbidity Index (CCI), race/ethnicity (categorized as white, black, Hispanic, or other [the “other” category included Asian, American Indian, and Alaska Native], Social Vulnerability Index (SVI), residential area (metropolitan vs non-metropolitan), cancer type, disease stage, Medicaid eligibility, and admission type (urgent vs elective).

The SVI is generated from census tract-level data and represents a composite measure of community vulnerability and resilience to external pressures.^18^ The study categorized SVI into tertiles, with the first tertile representing the least social vulnerability and the third tertile representing the highest social vulnerability.

Additionally, ICD-9/10 codes were used to identify any history of comorbid conditions associated with opioid misuse, such as substance abuse (excluding opioids) and mental health conditions.^19^ Mental health conditions included anxiety, depression, and psychotic disorders such as bipolar disorder and schizophrenia.

The primary outcome of interest was mortality within 181 to 365 days after index surgery. The secondary outcomes included hospital (inpatient and outpatient), or emergency department visits related to falls, respiratory events, or pain. Notably, patients who died during the year after surgery were not included in the secondary outcomes analysis.

A previously defined algorithm was used to capture fall-related injuries based on ICD-9/10 and Current Procedural Terminology codes from inpatient and emergency encounters.^20,21^ Moreover, previously validated diagnosis codes were used to identify hospital or emergency department visits for pain symptoms and respiratory events in claims data.^12,22^

### Statistical Analysis

Categorical variables are presented as frequencies and percentages, whereas continuous variables are reported as medians with interquartile ranges (IQRs). Continuous variables were compared using the Wilcoxon rank-sum test, whereas categorical variables were assessed using either the chi-square test or Fisher’s exact test as appropriate. Multivariable logistic regression was used to identify factors associated with postoperative NPOU development. Odds ratios (ORs) and 95 % confidence intervals (CIs) were reported. The multivariable analysis used patient-level factors including age, sex, CCI score, ethnicity, history of substance abuse or mental illness, SVI, residential area, cancer type, stage of disease, Medicaid eligibility, urgent index surgery, perioperative opioid use, and complications during the index hospitalization. Moreover, risk-adjusted Cox proportional hazard models were used to investigate the association between NPOU and survival time.

All statistical analyses were conducted using SAS 9.4 (SAS Institute, Cary, NC, USA). A *p* value lower than 0.05 was considered statistically significant.

## Results

### Baseline Characteristics

A total of 15,456 Medicare beneficiaries underwent gastrointestinal surgery for a malignant indication (hepatic [*n* = 288, 1.9%], pancreatic [*n* = 948, 6.1%], biliary duct [*n* = 459, 3.0%], colorectal [*n* = 13,761, 89.0%]). The median age was 75 years (IQR, 70–81 years). Most of the patients were females (*n* = 8435, 54.6%), had a CCI score of 2 or lower (*n* = 14,161, 91.6%), and lived in a metropolitan area (*n* = 12,682, 82.1%). Notably, 6.9% (*n* = 1071) of the patients had a history of substance abuse (other than opioids), and 14.2% (*n* = 2197) had a prior mental health condition.

A majority of the individuals lived in the West (*n* = 7504, 48.6%) or South (*n* = 5909, 38.2%), followed by the Midwest (*n* = 1085, 7.0%) and Northeast (*n* = 958, 6.2%) regions of the United States. Moreover, most of the individuals were white (*n* = 11,837, 76.6%), with smaller proportions of patients of black (*n* = 953, 6.2%), Hispanic (n=1179, 7.6%), or other (*n* = 1487, 9.6%) race/ethnicity.

Stage II disease (*n* = 5430, 35.1%) was the most common, followed by stage III (*n* = 4476, 29.0%), stage I (*n* = 4266, 27.6%), and stage IV (*n* = 1284, 8.3%) disease. Approximately one fifth of the patients were eligible for both Medicare and Medicaid (*n* = 3228, 20.9%), and about one half of the patients filled an opioid prescription during the perioperative period (*n* = 8779, 56.8%) (Table 1).

**Table 1:** Baseline characteristics of patients with and without a new persistent opioid use

Patient characteristics	Total (*n* = 15,456) *n* (%)	NPOU (*n* = 967) *n* (%)	No NPOU (*n* = 14,489) *n* (%)	*p* Value^a^
Age: years (range)	75 (70–81)	73 (69–78)	75 (71–81)	< 0.001
Sex Male Female	7021 (45.4) 8435 (54.6)	452 (46.7) 515 (53.3)	6569 (45.3) 7920 (54.7)	0.400
CCI ≤2 >2	14,161 (91.6) 1295 (8.4)	855 (88.4) 112 (11.6)	13,306 (91.8) 1183 (8.2)	< 0.001
Region Midwest Northeast South West	1085 (7.0) 958 (6.2) 5909 (38.2) 7504 (48.6)	86 (8.9) 89 (9.2) 324 (33.5) 468 (48.4)	999 (6.9) 869 (6.0) 5585 (38.5) 7036 (48.6)	< 0.001
Ethnicity White Black Hispanic Other	11,837 (76.6) 953 (6.2) 1179 (7.6) 1487 (9.6)	704 (72.8) 92 (9.5) 105 (10.9) 66 (6.8)	11,133 (76.8) 861 (5.9) 1074 (7.4) 121 (9.8)	< 0.001
History of substance abuse Mental health illness	1071 (6.9) 2197 (14.2)	112 (11.6) 160 (16.5)	959 (6.6) 2037 (14.1)	< 0.001 0.032
SVI Low Moderate High	512 (33.4) 5050 (32.9) 5162 (33.7)	263 (27.3) 343 (35.7) 356 (37.0)	4849 (33.8) 4707 (32.7) 406 (33.5)	< 0.001
Residential area Metropolitan Non-metropolitan	12,682 (82.1) 24 (17.9)	769 (79.5) 198 (20.5)	11,913 (82.2) 2576 (17.8)	0.034
Cancer type HCC PDAC Biliary duct CRC	288(1.9) 948(6.1) 459(3.0) 13,761(89.0)	26 (2.7) 94 (9.7) 40 (4.1) 807 (83.5)	262 (1.8) 854 (5.9) 419 (2.9) 12,954 (89.4)	< 0.001
Stage I II III IV	466 (27.6) 5430 (35.1) 4476 (29.0) 1284 (8.3)	151 (15.6) 305 (31.5) 326 (33.7) 185 (19.1)	4115 (28.4) 5125 (35.4) 4150 (28.6) 1099 (7.6)	< 0.001
Medicaid eligibility	3228 (20.9)	304 (31.4)	2924 (20.2)	< 0.001
Admission type Urgent Elective	3847 (24.9) 11,593 (75.1)	322 (33.4) 642 (66.6)	3525 (24.4) 10,951 (75.6)	< 0.001
Complications during index surgery	1613 (10.4)	135 (14.0)	178 (10.2)	< 0.001
Opioid fill during perioperative period	8779 (56.8)	786 (81.3)	7993 (55.2)	< 0.001
Average daily opioid dose	1.4 (0.8–3.0)	5.2 (2.8–11.0)	1.25 (0.8–2.1)	< 0.001
All-cause hospital visits	3946 (25.5)	482 (49.8)	3464 (23.9)	< 0.001
Hospital visits due to fall, respiratory symptoms, or pain	1385 (9.4)	130 (15.2)	1255 (9.0)	< 0.001
Mortality	694 (4.5)	111 (11.5)	583 (4.0)	< 0.001

*NPOU* new persistent opioid use, *CCI* charlson comorbidity index, *SVI* social vulnerability index, *HCC* hepatocellular carcinoma, *PDAC* pancreatic adenocarcinoma, *CRC* colorectal carcinoma

^a^Statistical significance denoted by *p* < 0.05

The patients who experienced NPOU were younger (73 years [range 69–78 years] vs 75 years [range, 71–81 years]), had a higher CCI score (CCI >2: NPOU [11.6%] vs no NPOU [8.2%]), and were more likely to be black (NPOU [9.5%] vs no NPOU [5.9%]) or Hispanic (NPOU [10.9%] vs no NPOU [7.4%]) (all *p* < 0.001). Moreover, individuals with a history of substance abuse (NPOU [11.6%] vs no NPOU [6.6%]) or mental illness (NPOU [16.5%] vs no NPOU [14.1%]), individuals residing in non-metropolitan areas (NPOU [20.5%] vs no NPOU [17.8%]), and the patients from moderate (NPOU [35.7%] vs no NPOU [32.7%]) or high (NPOU [37.0%] vs no NPOU [33.5%]) SVI areas were more likely to experience NPOU after surgery (all *p* < 0.001).

Furthermore, NPOU was more likely to occur among the patients who underwent surgery for stage III (NPOU [33.7%] vs no NPOU [28.6%]) or stage IV (NPOU [19.1%] vs no NPOU [7.6%]) disease (both *p* < 0.001). Stratification by cancer type showed that the patients who underwent surgery for hepatic (NPOU [2.7%] vs no NPOU [1.8%]), pancreatic (NPOU [9.7%] vs no NPOU [5.9%]), or biliary tract (NPOU [4.1%] vs no NPOU [2.9%]) cancer were more likely to experience NPOU (all *p* < 0.001). Importantly, the patients who were prescribed perioperative opioids (NPOU [81.3%] vs no NPOU [55.2%]) or received a high postoperative opioid dose (average daily dose [morphine milligram equivalents (MME)]: 5.2 [range, 2.8–11.0] vs 1.25 [range, 0.8–2.1]) were more likely to experience NPOU (both *p* < 0.001) (Table 1).

### Incidence and Risk Factors for NPOU

The overall incidence of NPOU after a gastrointestinal cancer surgical procedure was 6.3% (*n* = 967). Multivariable analysis showed that the factors associated with higher odds of experiencing NPOU were a history of substance abuse (other than opioid abuse) (OR, 1.45; 95% CI, 1.14–1.84), Medicaid eligibility (OR, 1.88; 95% CI, 1.59–2.21), moderate SVI (OR, 1.26; 95% CI, 1.06–1.50), and urgent admission (OR, 1.31; 95% CI, 1.16–1.49). Furthermore, an increased likelihood of NPOU development was associated with an advanced disease stage at presentation (reference: stage I, stage II [OR, 1.53; 95% CI, 1.25–1.88], stage III [OR, 2.16; 95% CI, 1.76–2.25], stage IV [OR, 4.42; 95% CI, 3.51–5.82] disease) and the use of perioperative opioids (OR, 3.07; 95%, CI 2.59–3.63). The patients who underwent surgery for liver (OR, 1.57; 95% CI, 1.02–2.41) or pancreatic (OR, 1.76; 95% CI, 1.38–2.25) cancer had higher odds of experiencing NPOU than those who underwent resection of CRC (Table 2).

**Table 2:** Factors associated with new persistent opioid use (NPOU) development

Patient characteristics	OR of new persistent opioid use (95 % CI)
Sex	
Male Female	Ref 0.97 (0.84–1.12)
CCI	
≤2 >2	Ref 1.25 (0.96–1.63)
Ethnicity	
White Black Hispanic Other	Ref 1.12 (0.87–1.43) 1.15 (0.91–1.46) 0.56 (0.42–0.74)^a^
SVI	
Low Moderate High	Ref 1.26 (1.06–1.50)^a^ 1.13 (0.95–1.35)
Residential area	
Non-metropolitan Metropolitan	Ref 0.88 (0.74–1.05)
Cancer type	
CRC HCC PDAC Biliary duct	Ref 1.57 (1.02–2.41)^a^ 1.76 (1.38–2.25)^a^ 1.36 (0.96–1.92)
Stage	
I II III IV	Ref 1.53 (1.25–1.88)^a^ 2.16 (1.76–2.65)^a^ 4.42 (3.51–5.82)^a^
Medicaid eligibility	1.88 (1.59–2.21)^a^
History of substance abuse	1.45 (1.14–1.84)^a^
History of mental illness	1.04 (0.86–1.25)
Admission type	
Elective Urgent	Ref 1.31 (1.16–1.49)^a^
Perioperative opioid use	3.07 (2.59–3.63)^a^

*OR* odds ratio, *CI* confidence interval, *CCI* charlson comorbidity index, *SVI* social vulnerability index, *CRC* colorectal carcinoma, *HCC* *hepatocellular carcinoma*, *PDAC* pancreatic adenocarcinoma

^a^Statistical significance denoted by *p* <0.005

### Long-term Health Care Outcomes and Survival

In the 6 to 12 months after gastrointestinal cancer surgery, patients with NPOU were more likely to visit the hospital or emergency department due to falls, respiratory symptoms, or pain (15.2% vs 9.2%; *p* < 0.001). In the multivariable analysis, after adjustment for clinical and demographic covariates, NPOU was associated with 45% higher odds of a hospital or emergency department visit due to falls, respiratory symptoms, or pain (OR, 1.45; 95% CI, 1.18–1.78; Table 3).

**Table 3:** Multivariable regression analysis examining the association between new persistent opioid use (NPOU) and long-term health outcomes^a^

Outcome	No NPOU	NPOU
Hospital visits due to falls, respiratory symptoms, or pain, (OR; 95% CI)	Ref.	1.45 (1.18–1.78)
1 year mortality, (HR; 95% CI)	Ref.	2.15 (1.74–2.66)

*OR* odds ratio, *HR* hazard ratio

^a^Model adjusted for patient age, sex, CCI score, ethnicity, history of substance abuse or mental illness, SVI, residential area, cancer type, stage of disease, Medicaid eligibility, urgent index surgery, perioperative opioid use, and complications during the index surgery

The 1 year overall survival rate was 60.8% (95% CI, 60.0–61.6%). The patients who experienced NPOU (42.3%; 95% CI, 39.2–45.4%) had a shorter median overall survival than the patients who did not experience NPOU (62.0%; 95% CI, 61.2–62.8%).

**Fig. 2:**
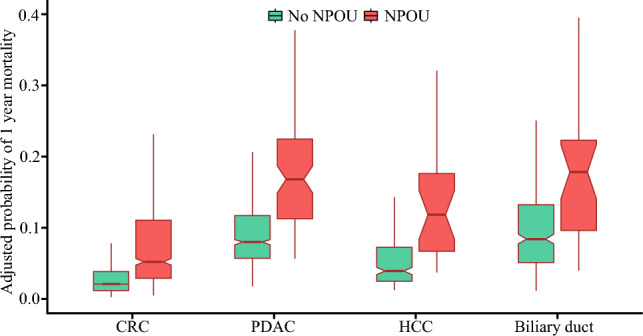
Box plot demonstrating the adjusted risk of 1 year mortality among patients with and without new persistent opioid use (NPOU)

In the risk-adjusted analysis, the patients who experienced NPOU had more than a twofold risk of death at 1 year than those who did not experience NPOU (1 year: hazard ratio [HR], 2.15; 95% CI, 1.74–2.66; *p* < 0.001; Fig. 2). Moreover, 578 patients (3.7%) continued using prescription opioids beyond 180 days. Notably, there was a dose-related relationship between opioids and mortality. In particular, compared with the patients who did not experience NPOU, the risk of mortality increased in a stepwise fashion among those with NPOU for 6 months (HR, 1.89; 95% CI, 1.36–2.63) and those with NPOU for 1 year (HR, 2.33; 95% CI, 1.81–3.01) (Table S1).

### Sensitivity Analysis

A sensitivity analysis was performed to assess the risk of hospital visits and mortality stratified by cancer type and disease stage. The patients who experienced NPOU had higher odds of hospital visits regardless of disease stage (ref: no NPOU; early stage [OR, 1.50; 95% CI, 1.07–1.96], advanced stage [OR, 1.48; 95% CI, 1.12–1.97). Furthermore, the adjusted risk of mortality was higher among the patients who experienced NPOU, irrespective of cancer type (ref: no NPOU; HPB [OR, 2.13; 95% CI, 1.46–3.10], CRC [OR, 2.14; 95% CI, 1.65–2.76) or disease stage (ref: no NPOU; early stage [OR, 1.74; 95% CI, 1.16–2.60], advanced stage [OR, 2.30; 95% CI, 1.80–2.95) (Table S2).

## Discussion

As the number of annual opioid overdose-related deaths in the United States continues to rise, curbing opioid misuse remains a persistent public health crisis.^1,2^ Notably, perioperative opioid use has been identified as a major contributor to the current opioid epidemic because more than 35% of all opioid prescriptions are for perioperative use.^23^ In turn, perioperative opioids function as a potential gateway for continued opioid misuse when patients consistently request refills beyond the anticipated time frame for pain resolution, resulting in NPOU.^16^ Notably, patients undergoing oncologic procedures may be vulnerable to the development of NPOU, as they are commonly prescribed higher doses of opioids for extended periods.^24^

Nonetheless, the incidence and long-term health care outcomes of NPOU among patients undergoing gastrointestinal cancer surgery remain ill-defined. Therefore, the current study was important because it addressed this significant literature gap by using nationally representative data to evaluate long-term clinical outcomes among patients who experienced NPOU after gastrointestinal cancer surgery. Notably, NPOU was associated with higher morbidity and mortality because the odds of hospital or emergency department visits related to falls, respiratory symptoms, or pain increased by 45%. Moreover, patients who experienced NPOU had more than a twofold increase in the risk of death 1 year after surgery.

Perioperative pain control is a crucial aspect of surgical care, and patients typically receive opioids only for short-term breakthrough pain control.^25^ Nonetheless, prior studies have reported that patients may continue using opioids beyond the intended duration.^13,26^ As such, NPOU use has been increasingly recognized as a common postoperative complication.^13,26^ For instance, Roberts et al.^26^ studied elderly female patients undergoing breast cancer surgery and reported that 2.8% of the patients experienced NPOU postoperatively. Similarly, Brummett et al.^13^ reported an NPOU incidence of 6.5% after major surgery, similar to the 6.3% incidence rate noted in the current study. Notably, the incidence of NPOU in our cohort was higher than that reported for patients who underwent surgery for breast cancer.^26^ This difference may have been attributable to the aggressive nature of gastrointestinal cancers, which often require extensive surgery and pain control with higher doses of opioids for an extended duration, thereby increasing the risk for the development of NPOU.^27^

Importantly, the current study highlighted that individuals with a history of substance abuse, Medicaid eligibility, or high social vulnerability were at a higher risk for the development of NPOU. Notably, these individuals may face several social, health, and economic stressors that contribute to NPOU.^28,29^ Specifically, individuals residing in areas with high SVI have poor health literacy, inadequate access to health care facilities, and multiple comorbid conditions requiring pain control that can result in continued opioid use even when not medically necessary.^28,29^ Consequently, NPOU is a common event among vulnerable patients undergoing high-risk surgery for a malignant indication. Therefore, before prescribing opioids, clinicians must screen for social risk factors that may contribute to the development of postoperative NPOU. In turn, referral to appropriate counseling services may be necessary to highlight the potential dangers associated with prolonged use of opioids and reduce the risk of NPOU.

Persistent use of opioids contributes to an additional health care burden, with greater health care use and expenditures in the postoperative period.^12,30^ Prior studies have demonstrated that perioperative opioid prescription carries a dose-dependent risk of falls and related injuries, especially in the elderly population.^20^ Additionally, patients with continuous opioid use after surgery are at risk of respiratory depression and other respiratory complications.^31^ Similarly, the current study noted that Medicare beneficiaries with NPOU had 45% greater odds of hospital or emergency department visits due to falls, pain symptoms, or respiratory events than individuals without NPOU. These findings were in line with a study by Santosa et al.^12^ that observed a similar pattern of increased hospital visits due to pain among patients with NPOU after common general surgical procedures.

Although opioids are commonly used as painkillers, there is a lack of evidence demonstrating their long-term effectiveness.^32^ Opioid-induced hyperalgesia during the acute perioperative period is well-documented, and more recently, this phenomenon has been observed among individuals with prolonged opioid exposure.^32,33^ In the current study, the reasons for frequent hospital visits due to pain among the patients with NPOU were likely multifactorial, although opioid-induced hyperalgesia may have played an important role.^34^ Importantly, the current data also demonstrated that the patients with NPOU were associated with a greater risk of mortality 1 year after surgery. This finding may have been attributable to several underlying risk factors and may not have been solely due to NPOU. For example, the patients who experienced NPOU were more likely to have a history of prior substance abuse (other than opioids), to reside in areas with greater socioeconomic vulnerability, and to present with late-stage disease. These factors have been associated previously with poor postoperative outcomes and a higher risk of mortality.^35–37^ Nonetheless, the patients with NPOU did face an elevated risk of confounder-adjusted 1 year mortality. Taken together, these factors show that NPOU poses a major health care challenge that necessitates prompt and effective interventions to alleviate the extensive health care burden on patients with gastrointestinal cancer.

The results of the current study underscore the importance of preoperative risk stratification and comprehensive perioperative pain management plans to prevent NPOU. In the preoperative setting, patients should be screened for NPOU risk factors, and if opioids are prescribed, close monitoring is needed.^38^ For instance, the Toronto General Hospital introduced transitional pain services to identify patients at risk for opioid misuse and decrease the risk of misuse through education, the development of a personalized pain management plan, and close follow-up evaluation for weeks to months after surgery.^39^ Moreover, in the postoperative period, surgical pain may be managed through multimodal analgesia, with a focus on non-opioid medications such as acetaminophen, non-steroidal anti-inflammatory drugs, gabapentin, and ketamine.^40–42^ In fact, a combination of non-opioid analgesics can provide equivalent pain relief among patients with cancer.^43^ Furthermore, Razi et al.^44^ reported that an enhanced recovery after surgery (ERAS) protocol was beneficial in reducing inpatient opioid requirements and the subsequent development of NPOU among patients with lung cancer.

There is a need for opioid stewardship programs that focus on prescription monitoring, patient education, and health care provider training.^40^ In line with this, the Massachusetts General Hospital implemented several multidisciplinary interventions, including opioid guidelines for common surgical procedures, health care provider-focused posters in all surgical units, patient opioid awareness brochures, and educational seminars for residents, nurses, and advanced practice providers.^45^ As a result, opioid prescription doses decreased significantly without the need for refills.^45^

In general, preventing morbidity and mortality associated with NPOU requires a multifaceted approach.^40^ This approach should encompass cancer-specific opioid prescription guidelines, multimodal analgesia, frequent monitoring, increased awareness among patients regarding opioids, and health care provider education.^40^

The current study should be interpreted considering several limitations. The use of a large national database in the study was a notable strength. However, the retrospective design and dependence on an administrative database introduced the potential for residual confounding bias. Additionally, administrative datasets may be subject to coding errors and inaccurate data input. The study sample consisted of patients 65 years of age or older with Medicare coverage, which may have limited the generalizability of the results to younger patients and those with private insurance. Substance use disorders and mental health conditions are generally underreported in the SEER database. Therefore, beneficiaries identified with a substance use disorder or mental health condition may not be a true representation of the general population. Furthermore, SEER-Medicare data may have inadequate sensitivity to measure substance abuse and mental health disorders. Due to database limitations, a granular assessment of other painful conditions and mood disorders (e.g., severity of impairment or symptoms) that may be driving persistent use could not be examined. Medicare data do not provide details about the reason for opioid prescriptions. Moreover, pharmacy claims data provide information only on opioid fills, whereas patients may fill opioid prescriptions but choose not to use them. Prospective data are needed for a better understanding of specific patient-level risk factors and underlying causes associated with the development of NPOU.

In conclusion, 1 in 15 patients undergoing surgery for gastrointestinal cancer experienced NPOU postoperatively. As an additional health care burden, NPOU is associated with a greater likelihood of mortality and hospital visits due to falls, respiratory complications, and pain symptoms. These findings underscore the importance of preoperative screening, patient opioid awareness, use of non-opioid analgesics, and close monitoring of opioid prescriptions in the postoperative period.

### Supplementary Information

Below is the link to the electronic supplementary material.

Supplementary file1 (DOCX 13 kb)

## Data Availability

The data for this study were obtained from the linked SEER-Medicare database. There are restrictions to the availability of this data, which is used under license for this study. Data can be accessed with permission from the National Cancer Institute and Center for Medicare and Medicaid Services.
