# Supplementary material for: Long-term Health Outcomes of New Persistent Opioid Use After Gastrointestinal Cancer Surgery
Source: Ann Surg Oncol. 2024 May 18;31(8):5283–92. doi: 10.1245/s10434-024-15435-1 (PMC11236845; doi:10.1245/s10434-024-15435-1)
Supplement: Supplementary file 1 — Supplementary file1 (DOCX 13 kb) [file 10434_2024_15435_MOESM1_ESM.docx]

**Supplementary Table 1:** Multivariable regression analysis examining the association between duration of new persistent opioid use and mortality.

| **Duration of NPOU** | **HR (ref: no NPOU)** | **95% CI** |
| --- | --- | --- |
| 6 months | 1.89 | 1.36 – 2.63 |
| 1 year | 2.33 | 1.81 – 3.01 |

^NPOU, New persistent opioid use: HR, hazard ratio; HR, Hazard ratio^

**Supplementary Table 2:** Multivariable regression analysis examining the association between new persistent opioid use and long-term health outcomes (ref: no NPOU).

| **Patient characteristics** | **Hospital visits**  **(OR, 95% CI)** | **Mortality**  **(HR, 95% CI)** |
| --- | --- | --- |
| Cancer type  HPB  Colorectal | *  1.53 (1.23 – 1.90) | 2.13 (1.46 – 3.10)  2.14 (1.65 – 2.76) |
| Stage  Low (I & II)  High (III & IV) | 1.50 (1.07 – 1.96)  1.48 (1.12 – 1.97) | 1.74 (1.16 – 2.60)  2.30 (1.80 – 2.95) |

^NPOU, New persistent opioid use; OR, Odds ratio; HR, Hazard ratio; HPB, Hepatopancreatobiliary^

^* Calculation was not possible due to sample size limitations.^
